# Discovery of Immunoproteasome Inhibitors Using Large-Scale Covalent Virtual Screening

**DOI:** 10.3390/molecules24142590

**Published:** 2019-07-16

**Authors:** Andrea Scarpino, Dávid Bajusz, Matic Proj, Martina Gobec, Izidor Sosič, Stanislav Gobec, György G. Ferenczy, György M. Keserű

**Affiliations:** 1 Medicinal Chemistry Research Group, Research Centre for Natural Sciences, Hungarian Academy of Sciences, Magyar tudósok krt. 2, H-1117 Budapest, Hungary; 2 Faculty of Pharmacy, University of Ljubljana, Aškerčeva cesta 7, SI-1000 Ljubljana, Slovenia

**Keywords:** immunoproteasome, covalent inhibitor, virtual screening, β5i selective inhibitor

## Abstract

Large-scale virtual screening of boronic acid derivatives was performed to identify nonpeptidic covalent inhibitors of the β5i subunit of the immunoproteasome. A hierarchical virtual screening cascade including noncovalent and covalent docking steps was applied to a virtual library of over 104,000 compounds. Then, 32 virtual hits were selected, out of which five were experimentally confirmed. Biophysical and biochemical tests showed micromolar binding affinity and time-dependent inhibitory potency for two compounds. These results validate the computational protocol that allows the screening of large compound collections. One of the lead-like boronic acid derivatives identified as a covalent immunoproteasome inhibitor is a suitable starting point for chemical optimization.

## 1. Introduction

Targeted covalent inhibitors form chemical bonds with protein residues. Their potential advantages over noncovalent inhibitors are high potency, strong, often irreversible binding, long duration of action, and lower sensitivity to pharmacokinetic parameters [1,2]. The identification and the optimization of covalent inhibitors, however, require special considerations and methods typically not present in more conventional noncovalent drug discovery. This also holds for computational approaches supporting drug discovery efforts. The proper description of covalent bond formation poses particular challenges for computational methods. In noncovalent hit identification, virtual screening, especially structure-based virtual screening, has proven to be a successful tool to efficiently investigate large chemical libraries with 10^4^–10^5^ compounds [3]. Structure-based virtual screening tools have also been developed for covalent inhibitors, however, their limited throughput is not always coupled with improved quality results [4], and their prospective application to large compound collections is rare [5,6,7]. One of the limitations of covalent docking tools is the improper description of the conformational space available for a ligand covalently bound to the protein. Correctly accounting for the interplay between bonded and non-bonded interactions in the neighborhood of the newly formed covalent bond is highly challenging, and anchoring the covalent ligand at the reaction site does not necessarily improve binding mode predictions. Indeed, it was found that noncovalent docking tools can be applied to the docking of compounds forming covalent bonds with little loss of accuracy compared to dedicated covalent docking tools [4]. Taking into account the significantly higher throughput of noncovalent docking and scoring, it is appealing to build a multistep virtual screening protocol, where noncovalent docking is used to filter a large number of compounds, and then covalent docking is only applied to a limited number of pre-screened ligands. Such a procedure reflects the binding process of covalent compounds with the initial formation of the noncovalent complex before the covalent bond is formed. In the present contribution, we propose a three step protocol that starts with a noncovalent docking-scoring step to significantly reduce the number of ligands that are input into two consecutive covalent docking steps; a higher throughput and less accurate scheme is followed by a more involved calculation with higher precision, as is detailed later.

We applied a virtual screening-based hit searching procedure to identify inhibitors for the chymotrypsin-like (β5i) subunit of the 20S immunoproteasome [8] that represents a part of the ubiquitin-proteasome system responsible for the degradation of proteins. The 20S proteasome (the core particle, CP) has three active subunits, namely β1 (caspase-like), β2 (trypsin-like), and β5 (chymotrypsin-like) subunits, each having different substrate specificities. β1, β2, and β5 are replaced by the corresponding β1i, β2i, and β5i subunits in the immunoproteasome. While the constitutive proteasome (cCP) is expressed in all eukaryotic cells, the immunoproteasome (iCP) is mainly induced during disease processes [9]. Proteasome inhibitors were found to be effective in cancer therapy [10]; for example, bortezomib [11], a covalent drug, was approved by the Food and Drug Administration (FDA) for the treatment of multiple myeloma and mantle cell lymphoma. Although several inhibitors of both proteasomes have been reported [12,13], selective nonpeptidic inhibitors of the iCP are still scarce [14,15,16,17,18]. Selective inhibition of the iCP is expected to cause fewer side effects, while nonpeptidic inhibitors are expected to have better pharmacokinetic properties than most currently available inhibitors with peptide-like scaffold [12,19].

We decided to search for iCP inhibitors with boronic acid warhead attached to a nonpeptidic scaffold. The reasons for that are the recent studies, which showed that inhibition of β5i is imperative to achieve beneficial effects in autoimmune diseases [20,21]. We performed structure-based virtual screening against the β5i subunit of the human iCP with the perspective to develop β5i-selective covalent inhibitors. Boronic acid derivatives are inhibitors of various proteins, including serine proteases, β-lactamases, histone deacetylases, and the proteasomes, and for some of these targets, the covalent attachment to serine or threonine residues has been shown [22]. Nonpeptidic inhibitors are expected to have beneficial pharmacokinetic profile and boronic acid derivatives without nearby amide nitrogen can avoid bioactivation [23]. Therefore, validated hits of the virtual screening campaign are potential chemical starting points for developing selective iCP inhibitors with improved properties.

## 2. Results

**Figure 1:**
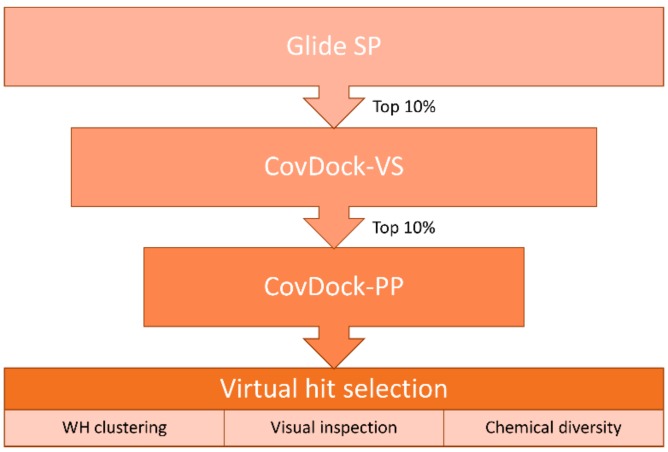
Hierarchical protocol used in the virtual screening of the boronic acid library.

We compiled a virtual library of compounds equipped with a boronic acid group to label the catalytic Thr1 with the same mechanism displayed by bortezomib. Boronic acids were collected from the ZINC [24] and the eMolecules [25] databases and then filtered for desirable molecular properties: number of heavy atoms (min. 10, max. 30), rings (min. 1, max. 5), rotatable bonds (≤10) and chiral centers (≤3), as well as atom types allowed in the structure (C, H, O, N, S, B, P, F, Cl, Br, I). This led to a library of over 104,000 unique ligands displaying the range of molecular descriptors shown in Figure S1. The use of state-of-the-art dedicated covalent docking tools allows modeling of the binding mode of covalent ligands. CovDock, the covalent docking program developed by Schrödinger, mimics the multi-step binding process of covalent modifiers by simulating both pre- and post-reactive states. It allows users to choose between a “pose prediction” (also named “Lead optimization”, from now on referred to as CovDock-PP) [26]) and a “virtual screening” mode (CovDock-VS) [27]. While the former is designed to predict accurate binding modes via more demanding simulations, the latter allows screening of larger libraries [28,29,30,31] by efficiently decreasing the number of steps in the protocol. However, even CovDock-VS has a throughput (≈15 CPU minutes/ligand) not compatible with the size of our virtual compound collection. Therefore, we designed a hierarchical virtual screening protocol to progressively reduce the number of compounds to be modeled with more demanding and therefore more accurate computational tools (Figure 1).

First, the library was screened by using Glide SP [32]. This noncovalent docking protocol enables us to efficiently screen hundreds of thousands of compounds, but it does not allow to model the warhead geometry in the bound form. To partially overcome this limitation, the Glide SP docking was performed by using the following conditions: 1) the targeted Thr1 was mutated to alanine in order to extend the sampling space available for the boronic acid group at the reaction site; 2) a positional constraint was set for the electrophile in order to filter out compounds not able to place it in the proximity of the targeted nucleophile. This was not only inspired by the fact that the protocol in CovDock starts with a constrained noncovalent docking into a protein structure whose reactive residue is mutated to alanine, but it is also supported by previous studies showing that such an approach guarantees high chances of accurate binding mode predictions [4].

Next, compounds ranked in top 10% by docking score (around 10,000 unique ligands) were moved forward to the CovDock-VS [27]. In this step, the screening was performed on the original protein structure with no mutation of the reactive Thr1. This residue is indeed required by CovDock, since the program uses a SMARTS pattern to match the reactive groups on both ligands and protein and then performs the covalent bond formation according to the selected reaction type (in this case, “Boronic Acid Addition”). Virtual screening by CovDock-VS could produce a protein-bound docking pose for around half of the input structures, thus further supporting the use of a faster noncovalent protocol as a filter to reduce the number of molecules to be subjected to more demanding covalent docking simulations.

Additionally, in this case, unique ligands ranked in top 10% by docking score were screened with the pose prediction mode of CovDock [26]. Contrarily to the virtual screening mode, CovDock-PP keeps a large number of clustered complexes that are then refined via the Prime energy model [33]. Furthermore, ligands are ranked by means of a dedicated scoring function named “affinity score” instead of the default GlideScore used by CovDock-VS. This score estimates the apparent affinity of covalent binders by averaging the docking score calculated for both the pre- and the post-reaction forms, thus trying to capture all the key steps of the binding process of covalent ligands.

The top 80 unique ligands ranked by affinity score were clustered in six groups according to the structure directly attached to the boronic acid warhead. Finally, visual inspection, chemical diversity, and supplier’s availability were used to select compounds among the clustered virtual hits. Priority was given to compounds with a binding mode similar to Ro19 [16], a β5i selective noncovalent inhibitor; 32 of the selected compounds were commercially available and were purchased (Table S1). Five of the tested compounds exhibited detectable inhibitory potency at 100 μM concentration (Table 1).

All these compounds have similar architecture and shape. The boronic acid headgroup is bound to either an aromatic or a saturated ring that is linearly attached to two or three additional rings. Compounds **1** and **2** with lowest residual enzyme activity (Table 1) were further investigated. Binding of these compounds to the β5i subunit of immunoproteasome was directly confirmed by microscale thermophoresis measurements (see Supplementary Data). In addition, with biochemical fluorescence-based assays, we determined that compounds **1** and **2** inhibit the β5i subunit of the iCP with IC_50_ values of 34 µM and 45 µM, respectively (Table 2).

**Figure 2:**
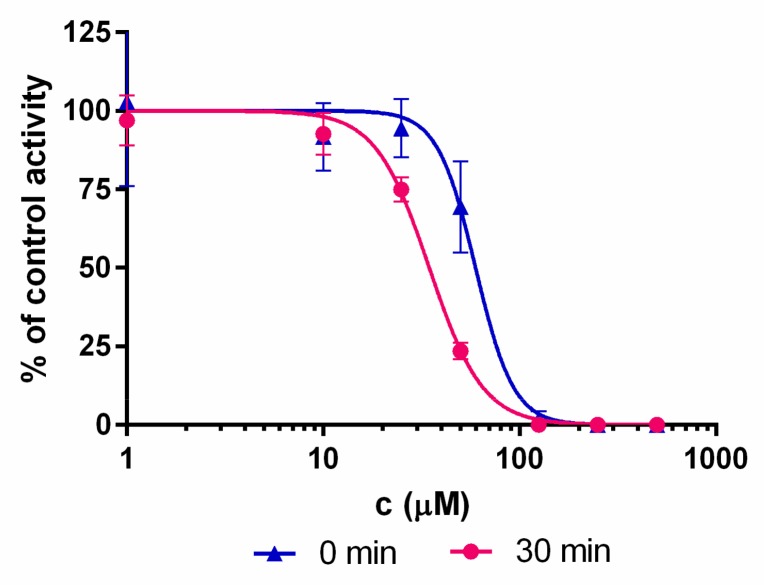
Representative shift of the IC_50_ curves for compound **1.** The shift to the left is characteristic for the time-dependent type of enzyme inhibition.

Boronic acids are expected to bind covalently and reversibly to the terminal Thr1 residue of the β5i subunit [34,35]. Covalent binding is typically associated with an appreciable reaction barrier that causes time-dependent inhibition. To investigate this point, inhibitory potency was measured with (30 min) and without pre-incubation of compounds and the iCP before the addition of the substrate. Both compounds **1** and **2** show time-dependent IC_50_ values (Figure 2 and Table 2), thus strongly indicating their covalent binding mechanism.

## 3. Discussion

In four of the active compounds shown in Figure 2, a pyrrolidine ring is attached to the boronic acid warhead, and only a single compound has an aromatic ring instead of the pyrrolidine. This is remarkable since aromatic boronic acids (25) are more represented than pyrrolidines (7) among the 32 compounds tested (Table S1). Moreover, the two compounds showing highest activity also have the boronic acid attached to a pyrrolidine ring. The preference of the pyrrolidine over the benzene ring in these covalent binders is in line with the higher reactivity of alkyl boronic acids compared to aromatic boronic acids [36].

**Figure 3:**
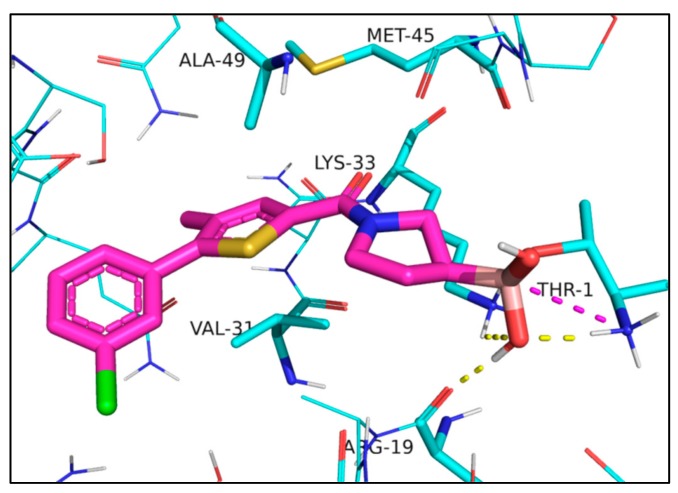
Interactions of compound **1** docked into iCP, Protein Data Bank (PDB) code: 5M2B [16]; **1** is represented as a thick tube with carbon atoms in magenta. Interacting residues discussed in the text are in a thin tube, and other residues are in a wire representation. Interactions are shown with dashed lines: ionic interactions in magenta and hydrogen bonds in yellow.

The docked compounds form covalent bond with the side chain of Thr1. In the post-reaction form, the negatively charged boron atom attracts the positively charged terminal Thr1 NH_3_^+^ group and the sidechain of Lys33. In addition, an OH-group attached to the boron atom accepts a hydrogen bond from the terminal NH_3_^+^ group of Thr1 and donates a hydrogen bond to the backbone carbonyl of Arg19. The three rings fit well into a predominantly hydrophobic pocket and form mainly van der Waals contacts with residues Lys33, Met45, Ala49, and Val31, the sidechain of the latter adopting a quasi-parallel position with the thiophene and the furan ring of **1** and **2**, respectively (Figure 3).

**Figure 4:**
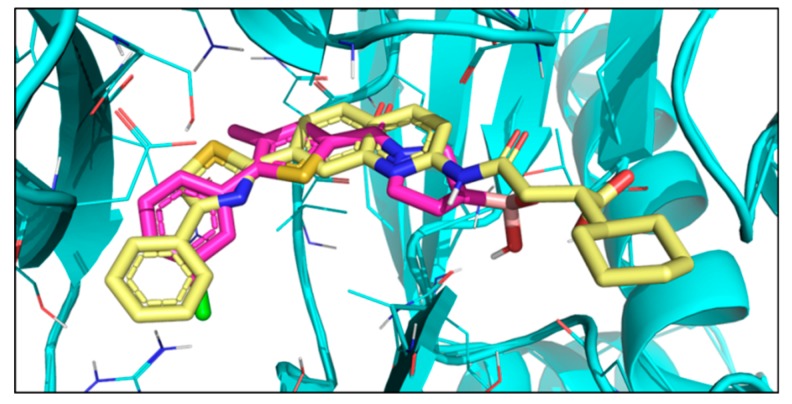
X-ray structure of Ro19 (yellow) (PDB code: 5M2B [16]) and docked pose of compound **1** (magenta).

The binding mode obtained from docking was compared to that of bortezomib [11], a covalent dipeptide boronic acid drug, and also to Ro19 [37], a nonpeptidic noncovalent inhibitor. The crystal structures of both compounds in complex with yeast 20S proteasome containing human β5i unit are available (bortezomib PDB code: 5L5F [38], Ro19 PDB code: 5M2B [16]). Ro19 and bortezomib bind differently and occupy quasi-orthogonal positions. The docked poses of compounds **1**–**5** are highly similar to each other and follow that of Ro19 (Figure 4). This is in line with our virtual hit selection process, where preference was given to Ro19 binding mode. This binding mode is assumed to be beneficial for achieving β5i selectivity. Ro19 exhibits an IC_50_ of 0.42μM for β5i that is almost 70-fold lower than the IC_50_ for β5, and this is mostly attributed to its binding into the S1 subpocket [16], where the different conformation of Met45 in β5i and β5 results in differently sized pockets [39]. Indeed, compounds **1** and **2** exhibit preference for iCP over cCP, as shown by their IC_50_ values obtained with 30 min pre-incubation time (Table 2).

Pyrrolidine boronic acids have a chiral center at the attachment of the boronic acid warhead to the pyrrolidine ring. However, the seven pyrrolidine boronic acid derivatives (as available from vendors and tested) are all racemic mixtures (Table S1). In contrast, the compound library subjected to virtual screening contains compounds with specified chiralities, and we observed that best scoring compounds include both R and S enantiomers. For example, compounds **1** and **2** as obtained from ZINC [24] (see Table S1 for ZINC codes) have opposite chirality. A comparison of docking poses of **1** and **2** shows that, in spite of the opposite chirality, these compounds are able to superimpose well and adopt highly similar binding modes (Figure S2).

## 4. Materials and Methods

### 4.1. General Chemistry Methods 

All compounds were purchased from Enamine Ltd., and they were used for biochemical screening as supplied. Compounds **1** and **2** were purified using column chromatography before performing analyses and assays. For flash column chromatography, Merck Silica Gel 60 (particle size 0.040–0.063 mm) was used. NMR spectra were recorded on a Bruker Avance III 400 MHz spectrometer (Bruker Corporation, Billerica, MA, USA) at 295 K. The chemical shifts (δ) are referenced to the deuterated solvent used and are reported in parts per million (ppm). The coupling constants (*J*) are given in Hz, and the splitting patterns are designated as follows: s, singlet; d, doublet; dd, double doublet; t, triplet; m, multiplet. Mass spectra were recorded using a Thermo Scientific Q Exactive Plus mass spectrometer (Thermo Fisher Scientific, Waltham, MA, USA). Analytical reversed-phase HPLC was performed on Thermo Scientific Dionex UltiMate 3000 UHPLC modular system (Thermo Fisher Scientific, Waltham, MA, USA) equipped with a photodiode array detector set to 254 nm. An Acquity UPLC^®^ BEH Phenyl Column (2.1 mm × 100 mm; 1.7 μm) was used and was thermostated at 40 °C, and flow rate was set to 0.3 mL/min. An eluent system of A (0.1% trifluoroacetic acid in H_2_O) and B (MeCN) was used with gradient elution: 0–10 min, 5% B → 95% B; 10–13 min, 95% B; 13–13.5 min, 95% B → 5% B.



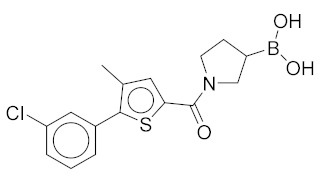



*(1-(5-(3-Chlorophenyl)-4-methylthiophene-2-carbonyl)pyrrolidin-3-yl)boronic Acid* (**1**). Starting amount 26 mg. Mobile phase for column chromatography was DCM/MeOH/AcOH = 20/1/0.1. ^1^H NMR (400 MHz, MeOD) δ 7.53–7.49 (m, 1H, Ar-H), 7.49–7.47 (m, 1H, Ar-H), 7.47–7.43 (m, 2H, 2 × Ar-H), 7.43–7.38 (m, 1H, Ar-H), 4.05–3.80 (m, 2H, CH_2_), 3.80–3.62 (m, 2H, CH_2_), 2.33 (s, 3H, CH_3_), 2.27–2.11 (m, 1H, CH), 1.99–1.78 (m, 2H, CH_2_). Electrospray ionization high-resolution mass spectrometry (ESI-HRMS) *m*/*z* calculated for C_16_H_18_O_3_NBClS [M + H]^+^ 350.0783, found 350.0779. Purity of the compound was 97.23%, as determined by HPLC.



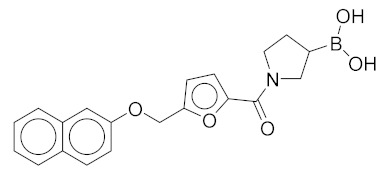



*(1-(5-((Naphthalen-2-yloxy)methyl)furan-2-carbonyl)pyrrolidin-3-yl)boronic Acid* (**2**). Starting amount 8 mg. Mobile phase for column chromatography was DCM/MeOH/AcOH = 9/1/0.1, ^1^H NMR (400 MHz, MeOD) δ 7.92 (dd, *J* = 8.5, 3.3 Hz, 1H, Ar-H), 7.74 (d, *J* = 8.0 Hz, 1H, Ar-H), 7.67 (d, *J* = 8.9 Hz, 1H, Ar-H), 7.64–7.18 (m, 1H, Ar-H), 7.44–7.36 (m, 1H, Ar-H), 7.29–7.23 (m, 1H, Ar-H), 7.14 (dd, *J* = 8.8, 1.6 Hz, 1H, Ar-H), 6.96 (t, *J* = 3.6 Hz, 1H, Ar-H), 6.10–6.02 (m, 1H, Ar-H), 4.47 (s, 2H, -OCH_2_-), 3.82–3.62 (m, 2H, CH_2_), 3.61–3.37 (m, 2H, CH_2_), 2.11–1.98 (m, 1H, CH), 1.83–1.56 (m, 2H, CH_2_). Due to presence of rotamers, when integrating only the large peaks, one aromatic signal was missing. The missing proton was integrated from the small peaks (7.64–7.18 ppm). ESI-HRMS m/z calculated for C_20_H_21_O_5_NB [M + H]^+^ 366.1507, found 366.1504. Purity of the compound was 91.02%, as determined by HPLC.

### 4.2. K_d_ Determination

Microscale thermophoresis measurements were conducted on a NanoTemper Monolith NT.115 device (NanoTemper Technologies GmbH, München, Germany). Human proteasome β5i subunit (PSMB8, ProSpec, cat. no: ENZ-600) was transferred from the vendor buffer to 20 mM phosphate buffer (pH 8.0) with 10% glycerol and was labeled with RED fluorescent NT-647-NHS dye (NanoTemper, cat. no: L001) following the suggested protocol. Ligand stocks were prepared in DMSO and diluted into the protein buffer, with final DMSO concentrations not exceeding 1%. Single-concentration binding measurements at 100 µM ligand concentration [with 0.65 µM protein, 40% light emitting diode (LED) power] revealed fluorescence changes significantly different from the DMSO control for both of the boronic acids as well as the known inhibitors PR-957 [40] and compound **42** in reference [14] (included for validation). Titration curves were acquired with serial 1:1 dilutions starting from 100 µM ligand concentration (with 0.13 µM protein, 80% LED power) (Figure S3). Each datapoint was acquired in duplicate.

### 4.3. Residual Activity Determination

The preliminary screening of compounds was performed at 100 μM final concentrations in assay buffer (0.01% SDS, 50 mM Tris-HCl, 0.5 mM EDTA, pH 7.4). To each compound, 0.2 nM human iCP (Boston Biochem, Inc., Cambridge, MA, USA) was added and incubated for 30 min at 37 °C. Afterwards, the reaction was initiated by the addition of Suc-LLVY-AMC (Bachem, Bubendorf, Switzerland) at 25 μM final concentration. The reaction progress was recorded on the BioTek Synergy HT microplate reader by monitoring fluorescence at 460 nm (λ_ex_ = 360 nm) for 90 min at 37 °C. The initial linear ranges were used to calculate the velocity and to determine the residual activity.

### 4.4. IC_50_ Determination

Compounds **1** and **2** were initially dissolved in DMSO and then added to black 96-well plates in the assay buffer (0.01% SDS, 50 mM Tris-HCl, 0.5 mM EDTA, pH 7.4) to obtain eight different final concentrations. The inhibitors were pre-incubated with 0.2 nM human iCP or 0.8 nM human cCP (both from Boston Biochem, Inc., Cambridge, MA, USA) at 37 °C for either 0 min or 30 min (for iCP) or for 30 min (for cCP) before the reaction was initiated by the Suc-LLVY-AMC substrate (Bachem, Bubendorf, Switzerland). The fluorescence was monitored at 460 nm (λ_ex_ = 360 nm) for 90 min at 37 °C. The progress of the reactions was recorded, and the initial linear ranges were used to calculate the velocity. IC_50_ values were calculated in Prism (GraphPad Software, CA, USA) and are means from at least three independent determinations.

### 4.5. Virtual Screening

Boronic acids were collected from the virtual databases ZINC and eMolecules, then filtered for specific properties as listed in Table 3.

This procedure led to a set of over 104,000 unique ligands displaying the range of molecular descriptors shown in Figure S1 (descriptors calculated by using RDKit [41]). LigPrep by Schrödinger was used to prepare the filtered set by generating three-dimensional (3D) conformations, tautomeric, and ionization states from SMILES codes at pH = 6–8 while retaining specified chiralities. The X-ray structure deposited in the Protein Data Bank (PDB) as 5M2B was used for the virtual screening. This entry corresponds to a yeast immunoproteasome with human β5i-immunoproteasome-substrate binding channel, including key residues of the adjacent β6 subunit in complex with a noncovalent inhibitor (Ro19). As the binding site is only defined by these two human subunits, all the others were removed together with ions and water molecules. The structure was prepared by means of the Protein Preparation Wizard [42,43] by Schrödinger, which was used to add hydrogen atoms, protonate residues at pH 7, refine the H-bond network, and to perform a restrained minimization. The receptor’s grid box required for docking calculations was centered on the corresponding co-crystallized ligand. As the first step of the cascade protocol, Glide SP noncovalent docking was used to screen the initial set of boronic acid derivatives. The catalytic residue Thr1 was mutated to Ala1 in order to increase the sampling space available for the ligands’ warhead. A positional constraint was set for the boron atom in the vicinity of Ala1-C_β_. These expedients were done to reflect the first steps of the covalent docking protocols developed by Schrödinger; indeed, both CovDock-VS and CovDock-PP start by mutating the reactive residue to alanine and then perform Glide docking with positional constraints (within 5Å from Ala-C_β_). Glide SP allowed us to rapidly screen a large library, such as the one collected in this work, in reasonable computational time (about 10 seconds/compound). The top 10% of docked compounds ranked by GlideScore were moved forward to covalent docking with CovDock-VS. For this purpose, the original unmutated protein structure was used to allow the covalent bond formation with Thr1. ”Boronic Acid Addition” was selected as the reaction type among the pre-defined ones listed by CovDock. Finally, the top 10% of docked compounds ranked by GlideScore (455 unique ligands) were subjected to the more demanding pose prediction module of CovDock. Docked compounds were ranked by ”affinity score”, and the 80 best scoring ligands were divided into six clusters according to the substructure directly attached to the boronic acid group in order to introduce variability in warheads’ reactivity. Finally, 32 virtual hits were selected [44,45] based on visual inspection, chemical diversity, and suppliers’ availability.

## 5. Conclusions

A virtual screening cascade is proposed that is able to screen hundreds of thousands of potential covalent binders. The basic novelty of the virtual screening protocol is the successive application of noncovalent and covalent docking steps that makes it possible to screen a high number of compounds not accessible for standard covalent docking tools. This protocol was successfully applied to identify covalent nonpeptidic boronic acid inhibitors of the β5i subunit of the iCP. The inhibitory potency of selected virtual hits was experimentally tested, and five compounds showed detectable activity. Microscale thermophoresis experiments were used to confirm binding to the β5i subunit, and the covalent mechanism was confirmed by observing time-dependent inhibition. The analysis of the binding mode of the docked compounds shows that they bind similarly to Ro19, a nonpeptidic and noncovalent β5i-selective ligand, and in a quasi-orthogonal position to peptidic covalent inhibitors. Validated hits occupy the S1 pocket, which was shown to confer selectivity with respect to the β5 subunit of the cCP. Compound **1**, identified by our hit finding procedure, is a suitable starting point for chemical optimization into a covalent, nonpeptidic, β5i-selective inhibitor of the iCP.

## Figures and Tables

**Table 1:** Biochemical assay results for the active virtual screening hits. To monitor the residual activity of the β5i subunit of the immunoproteasome (iCP), Suc-LLVY-AMC (Succinyl-Leu-Leu-Val-Tyr-7-amino-4-methylcoumarin) was used as the substrate.

Compound	Structure	Residual Activity (%) at 100 μM Compound
**1**	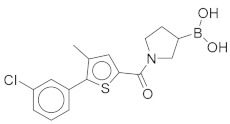	2 ± 4
**2**	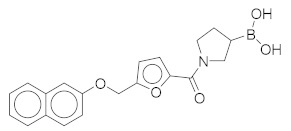	1 ± 4
**3**	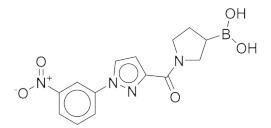	55 ± 9
**4**	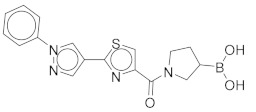	62 ± 2
**5**	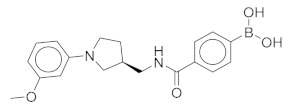	77 ± 15

**Table 2:** Dissociation constants (K_d_) by microscale thermophoresis and inhibitory potency (IC_50_) without pre-incubation and with 30 min pre-incubation of iCP and compounds and with 30-min pre-incubation of constitutive proteasome (cCP) and compounds. For IC_50_ determinations, Suc-LLVY-AMC was used as a substrate. As a positive control, PR-957 was used, and the experimentally determined IC_50_ value for inhibition of β5i subunit of the iCP was 0.018 ± 0.005 µM.

Compound	K_d_ (μM)	IC_50_ (μM) iCP		IC_50_ (μM) cCP
		Pre-Incubation Time: 0 min	Pre-Incubation Time: 30 min	Pre-Incubation Time: 30 min
1	22.4 ± 5.1	60 ± 7	34 ± 2	102 ± 1
2	41.1 ± 0.6	59 ± 6	45 ± 1	105 ± 5

**Table 3:** Molecular properties used to generate the virtual library of boronic acids.

Property Name	Property Value
Heavy atoms count	10–30
Rotatable bonds	≤ 10
Stereocenters	≤ 3
Rings count	1–5
Atoms allowed	C, H, O, N, S, B, P, F, Cl, Br, I

## Supplementary Materials

The following are available online, Figure S1: Distribution of molecular descriptors of the virtual library screened against the β5i subunit of the immunoproteasome; Figure S2: Superimposed docking poses of compounds **1** (magenta) and **2** (grey); Figure S3: Affinity measurement by microscale thermophoresis; Table S1: ZINC IDs, structures, and the results of biochemical characterization of the virtual screening hits.

## References

[1] De Cesco S., Kurian J., Dufresne C., Mittermaier A.K., Moitessier N. (2017). European Journal of Medicinal Chemistry Covalent inhibitors design and discovery. Eur. J. Med. Chem..

[2] Bauer R.A. (2015). Covalent inhibitors in drug discovery: From accidental discoveries to avoided liabilities and designed therapies. Drug Discov. Today.

[3] Lionta E., Spyrou G., Vassilatis D.K., Cournia Z. (2014). Structure-based virtual screening for drug discovery: principles, applications and recent advances. Curr. Top. Med. Chem..

[4] Scarpino A., Ferenczy G.G., Keserü G.M. (2018). Comparative Evaluation of Covalent Docking Tools. J. Chem. Inf. Model..

[5] London N., Miller R.M., Krishnan S., Uchida K., Irwin J.J., Eidam O., Gibold L., Cimermančič P., Bonnet R., Shoichet B.K. (2014). Covalent docking of large libraries for the discovery of chemical probes. Nat. Chem. Biol..

[6] Katritch V., Byrd C.M., Tseitin V., Dai D., Raush E., Totrov M., Abagyan R., Jordan R., Hruby D.E. (2007). Discovery of small molecule inhibitors of ubiquitin-like poxvirus proteinase I7L using homology modeling and covalent docking approaches. J. Comput. Aided. Mol. Des..

[7] Nnadi C.I., Jenkins M.L., Gentile D.R., Bateman L.A., Zaidman D., Balius T.E., Nomura D.K., Burke J.E., Shokat K.M., London N. (2018). Novel K-Ras G12C Switch-II Covalent Binders Destabilize Ras and Accelerate Nucleotide Exchange. J. Chem. Inf. Model..

[8] Marques A.J., Palanimurugan R., Matias A.C., Ramos P.C., Dohmen R.J. (2009). Catalytic Mechanism and Assembly of the Proteasome. Chem. Rev..

[9] Thibaudeau T.A., Smith D.M. (2019). A Practical Review of Proteasome Pharmacology. Pharmacol. Rev..

[10] Genin E., Reboud-Ravaux M., Vidal J. (2010). Proteasome inhibitors: recent advances and new perspectives in medicinal chemistry. Curr. Top. Med. Chem..

[11] Demo S.D., Kirk C.J., Aujay M.A., Buchholz T.J., Dajee M., Ho M.N., Jiang J., Laidig G.J., Lewis E.R., Parlati F. (2007). Antitumor Activity of PR-171, a Novel Irreversible Inhibitor of the Proteasome. Cancer Res..

[12] Huber E.M., Groll M. (2012). Inhibitors for the immuno- and constitutive proteasome: Current and future trends in drug development. Angew. Chemie Int. Ed..

[13] Ettari R., Zappalà M., Grasso S., Musolino C., Innao V., Allegra A. (2018). Immunoproteasome-selective and non-selective inhibitors: A promising approach for the treatment of multiple myeloma. Pharmacol. Ther..

[14] Sosič I., Gobec M., Brus B., Knez D., Živec M., Konc J., Lešnik S., Ogrizek M., Obreza A., Žigon D. (2016). Nonpeptidic Selective Inhibitors of the Chymotrypsin-Like (β5 i) Subunit of the Immunoproteasome. Angew. Chemie Int. Ed..

[15] Kasam V., Lee N.-R., Kim K.-B., Zhan C.-G. (2014). Selective immunoproteasome inhibitors with non-peptide scaffolds identified from structure-based virtual screening. Bioorg. Med. Chem. Lett..

[16] Cui H., Baur R., Le Chapelain C., Dubiella C., Heinemeyer W., Huber E.M., Groll M. (2017). Structural Elucidation of a Nonpeptidic Inhibitor Specific for the Human Immunoproteasome. ChemBioChem.

[17] Fan H., Angelo N.G., Warren J.D., Nathan C.F., Lin G. (2014). Oxathiazolones Selectively Inhibit the Human Immunoproteasome over the Constitutive Proteasome. ACS Med. Chem. Lett..

[18] Bosc E., Nastri J., Lefort V., Valli M., Contiguiba F., Pioli R., Furlan M., da Bolzani V.S., El Amri C., Reboud-Ravaux M. (2018). Piperlongumine and some of its analogs inhibit selectively the human immunoproteasome over the constitutive proteasome. Biochem. Biophys. Res. Commun..

[19] Kisselev A.F., Groettrup M. (2014). Subunit specific inhibitors of proteasomes and their potential for immunomodulation. Curr. Opin. Chem. Biol..

[20] Basler M., Lindstrom M.M., LaStant J.J., Bradshaw J.M., Owens T.D., Schmidt C., Maurits E., Tsu C., Overkleeft H.S., Kirk C.J. (2018). Co-inhibition of immunoproteasome subunits LMP2 and LMP7 is required to block autoimmunity. EMBO Rep..

[21] Johnson H.W.B., Lowe E., Anderl J.L., Fan A., Muchamuel T., Bowers S., Moebius D.C., Kirk C., McMinn D.L. (2018). Required Immunoproteasome Subunit Inhibition Profile for Anti-Inflammatory Efficacy and Clinical Candidate KZR-616 ((2*S*,3*R*)-*N*-((*S*)-3-(Cyclopent-1-en-1-yl)-1-((*R*)-2-methyloxiran-2-yl)-1-oxopropan-2-yl)-3-hydroxy-3-(4-methoxyphenyl)-2-((*S*)-2-(2-morpholinoacetamido)propanamido)propenamide). J. Med. Chem..

[22] Fu H., Fang H., Sun J., Wang H., Liu A., Sun J., Wu Z. (2014). Boronic acid-based enzyme inhibitors: a review of recent progress. Curr. Med. Chem..

[23] Li A.C., Yu E., Ring S.C., Chovan J.P. (2013). Boronic Acid-Containing Proteasome Inhibitors: Alert to Potential Pharmaceutical Bioactivation. Chem. Res. Toxicol..

[24] Sterling T., Irwin J.J. (2015). ZINC 15—Ligand Discovery for Everyone. J. Chem. Inf. Model..

[25] eMolecules. https://www.emolecules.com/.

[26] Zhu K., Borrelli K.W., Greenwood J.R., Day T., Abel R., Farid R.S., Harder E. (2014). Docking Covalent Inhibitors: A Parameter Free Approach To Pose Prediction and Scoring. J. Chem. Inf. Model..

[27] Toledo Warshaviak D., Golan G., Borrelli K.W., Zhu K., Kalid O. (2014). Structure-Based Virtual Screening Approach for Discovery of Covalently Bound Ligands. J. Chem. Inf. Model..

[28] Brogi S., Fiorillo A., Chemi G., Butini S., Lalle M., Ilari A., Gemma S., Campiani G. (2017). Structural characterization of Giardia duodenalis thioredoxin reductase (g TrxR) and computational analysis of its interaction with NBDHEX. Eur. J. Med. Chem..

[29] Muzzarelli K.M., Kuiper B., Spellmon N., Brunzelle J., Hackett J., Amblard F., Zhou S., Liu P., Kovari I.A., Yang Z. (2019). Structural and Antiviral Studies of the Human Norovirus GII.4 Protease. Biochemistry.

[30] Chowdhury S.R., Kennedy S., Zhu K., Mishra R., Chuong P., Nguyen A., Kathman S.G., Statsyuk A.V. (2019). Discovery of covalent enzyme inhibitors using virtual docking of covalent fragments. Bioorg. Med. Chem. Lett..

[31] Vasaturo M., Fiengo L., De Tommasi N., Sabatino L., Ziccardi P., Colantuoni V., Bruno M., Cerchia C., Novellino E., Lupo A. (2017). A compound-based proteomic approach discloses 15-ketoatractyligenin methyl ester as a new PPARγ partial agonist with anti-proliferative ability. Sci. Rep..

[32] (2018). Glide.

[33] (2018). Prime.

[34] Bull S.D., Davidson M.G., van den Elsen J.M.H., Fossey J.S., Jenkins A.T.A., Jiang Y.-B., Kubo Y., Marken F., Sakurai K., Zhao J. (2013). Exploiting the Reversible Covalent Bonding of Boronic Acids: Recognition, Sensing, and Assembly. Acc. Chem. Res..

[35] Groll M., Berkers C.R., Ploegh H.L., Ovaa H. (2006). Crystal structure of the boronic acid-based proteasome inhibitor bortezomib in complex with the yeast 20S proteasome. Structure.

[36] Marinaro W.A., Prankerd R., Kinnari K., Stella V.J. (2015). Interaction of Model Aryl- and Alkyl-Boronic Acids and 1,2-Diols in Aqueous Solution. J. Pharm. Sci..

[37] Alam M., Hawley R.C., Lynch S.M., Narayanan A. (2014). Substituted Thiazole Compounds. WO.

[38] Huber E.M., Heinemeyer W., de Bruin G., Overkleeft H.S., Groll M. (2016). A humanized yeast proteasome identifies unique binding modes of inhibitors for the immunosubunit β5i. EMBO J..

[39] Huber E.M., Basler M., Schwab R., Heinemeyer W., Kirk C.J., Groettrup M., Groll M. (2012). Immuno- and constitutive proteasome crystal structures reveal differences in substrate and inhibitor specificity. Cell.

[40] Muchamuel T., Basler M., Aujay M.A., Suzuki E., Kalim K.W., Lauer C., Sylvain C., Ring E.R., Shields J., Jiang J. (2009). A selective inhibitor of the immunoproteasome subunit LMP7 blocks cytokine production and attenuates progression of experimental arthritis. Nat. Med..

[41] RDKit: Open-Source Cheminformatics. Release 2019.03.1. http://www.rdkit.org.

[42] (2018). Protein Preparation Wizard.

[43] Madhavi Sastry G., Adzhigirey M., Day T., Annabhimoju R., Sherman W. (2013). Protein and ligand preparation: parameters, protocols, and influence on virtual screening enrichments. J. Comput. Aided. Mol. Des..

[44] (2019). JChem for Office 19.1.0.421, 2019.

[45] (2018). Maestro.

