# Supplementary material for: Discovery of Immunoproteasome Inhibitors Using Large-Scale Covalent Virtual Screening
Source: Molecules. 2019 Jul 16;24(14):2590. doi: 10.3390/molecules24142590 (PMC6680723; doi:10.3390/molecules24142590)
Supplement: Supplementary file 1 [file molecules-24-02590-s001.pdf]

Supplementary Material

Discovery of immunoproteasome inhibitors using large-scale covalent virtual screening

Andrea Scarpino [1], Dávid Bajusz [1], Matic Proj [2], Martina Gobec [2], Izidor Sosič [2], Stanislav Gobec [2], György G. Ferenczy [1], György M. Keserű [1,*]

[1] Medicinal Chemistry Research Group, Research Centre for Natural Sciences, Hungarian Academy of Sciences, Magyar tudósok krt. 2, H-1117 Budapest, Hungary
[2] University of Ljubljana, Faculty of Pharmacy, Aškerčeva cesta 7, SI-1000, Ljubljana, Slovenia
* Correspondence: keseru.gyorgy@ttk.mta.hu; Tel.: +36-1-3826-821

Contents

Figure S1. Distribution of molecular descriptors of the virtual library screened against the β5i subunit of the immunoproteasome. 2
Figure S2. Superimposed docking poses of compounds **1** (magenta) and **2** (grey) 3
Figure S3. Affinity measurement by microscale thermophoresis....................4
Table S1. ZINC IDs, structures, and the results of biochemical characterization of the virtual screening hits....................5
References....................10

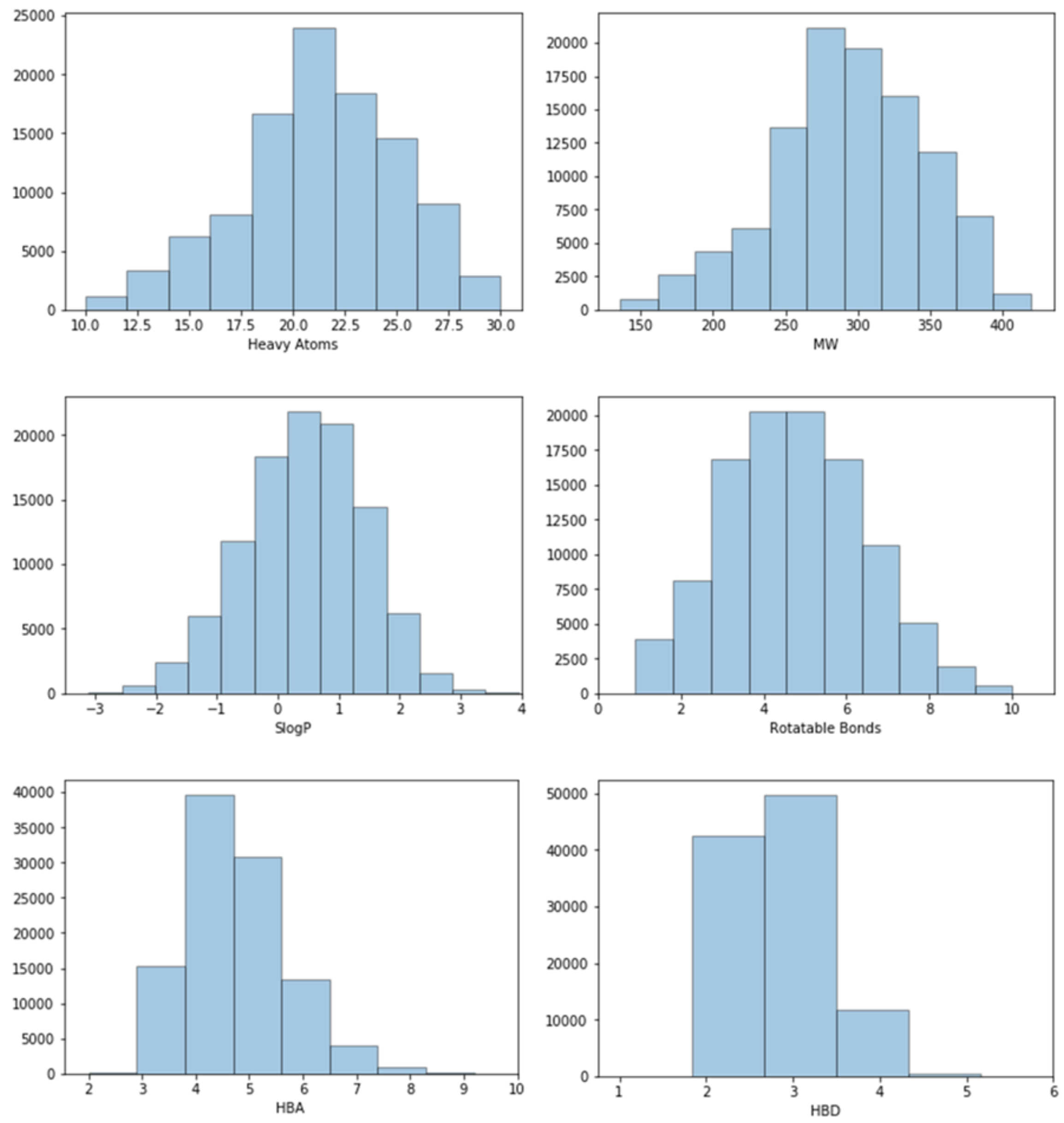

Figure S1. Distribution of molecular descriptors of the virtual library screened against the β5i subunit of the immunoproteasome. Descriptors were calculated by using RDKit.[1]

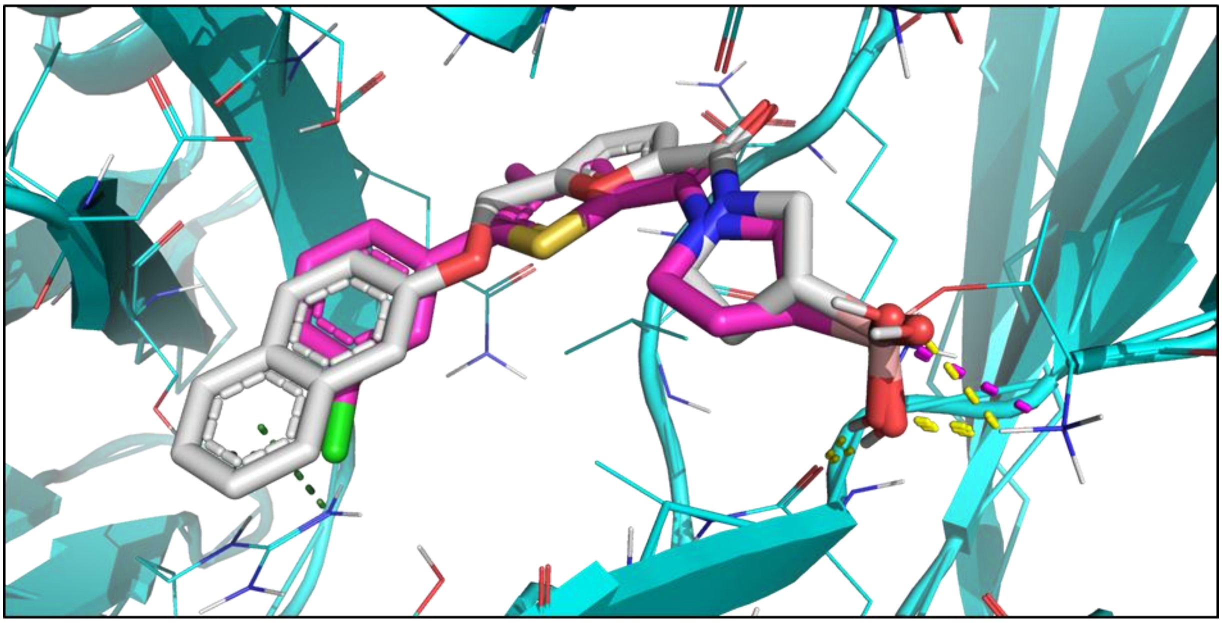

Figure S2. Superimposed docking poses of compounds **1** (magenta) and **2** (grey).

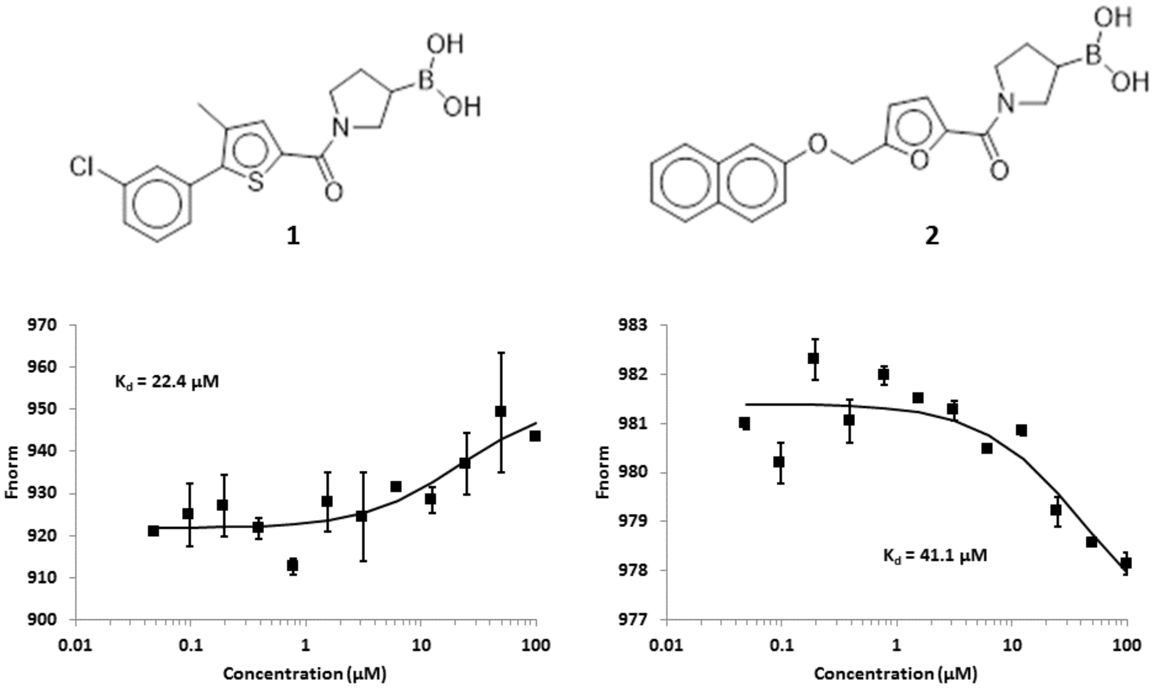

Figure S3. Affinity measurement by microscale thermophoresis

Table S1. ZINC IDs, structures, and the results of biochemical characterization of the virtual screening hits.

**ZINC ID (Number in main text)**	**Structure**	**RA[a] [%] (β5i) at 100 µM compound**
ZINC000585013431	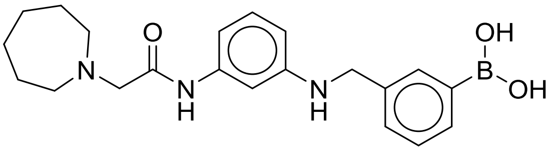	NA
ZINC000584965614	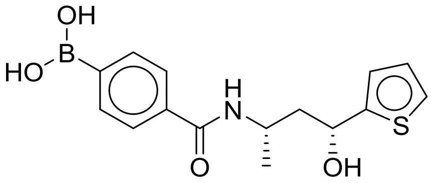	NA
ZINC000585006455	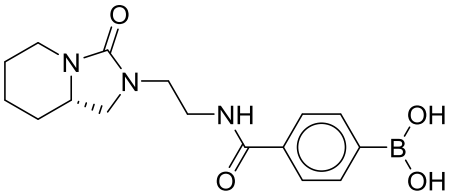	NA
ZINC000584940375 **(5)**	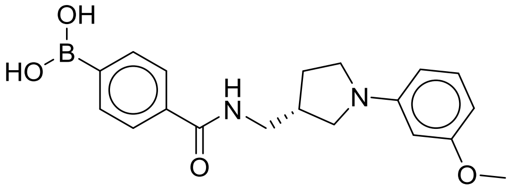	77±15
ZINC000584966354	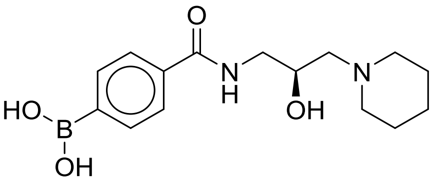	NA
ZINC000584998523	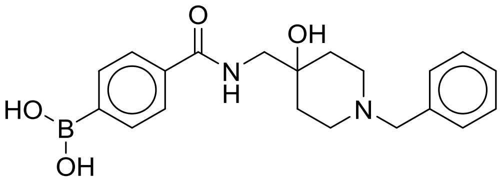	NA
ZINC000585008215	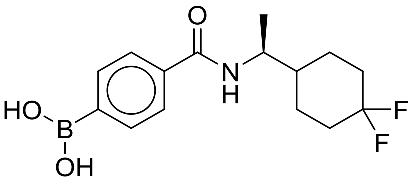	NA

ZINC000584933439	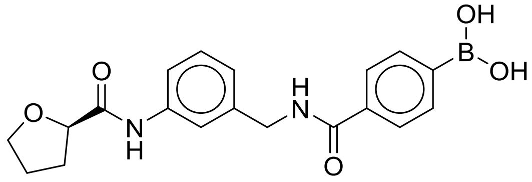	NA
ZINC000584962040	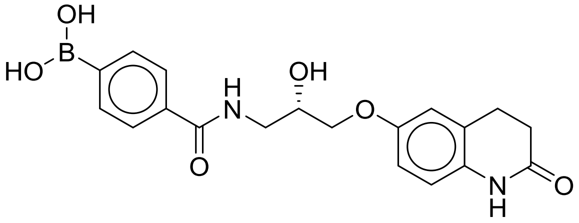	NA
ZINC000584987882	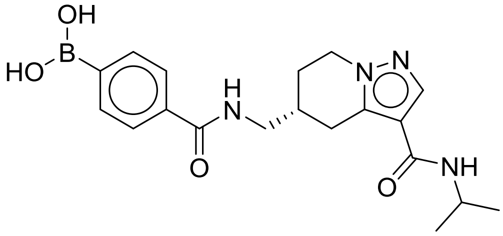	NA
ZINC000584963490	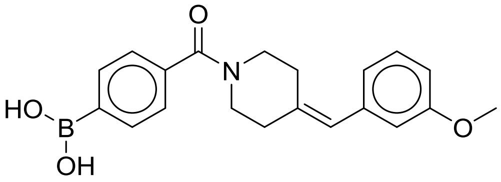	NA
ZINC000584975979	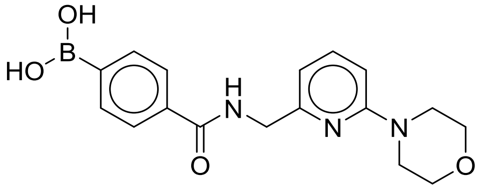	NA
ZINC000584927965	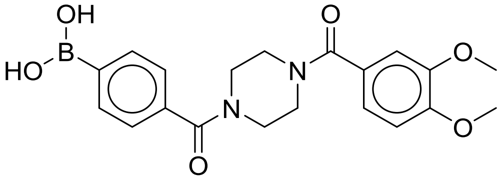	NA
ZINC000585009142	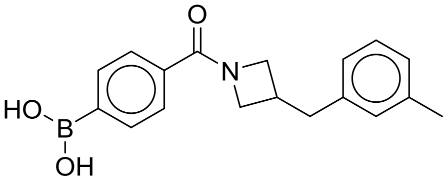	NA
ZINC000584929122	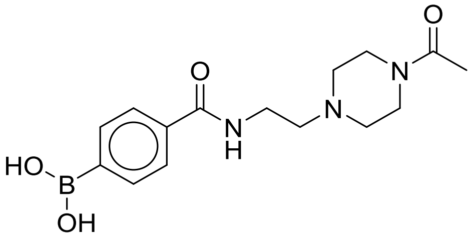	NA

ZINC000584982993	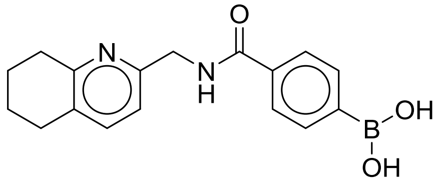	NA
ZINC000585009673	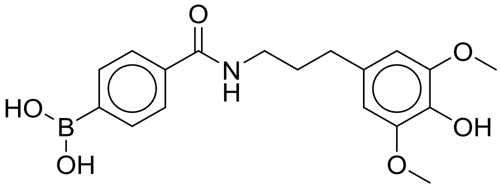	NA
ZINC000584976792	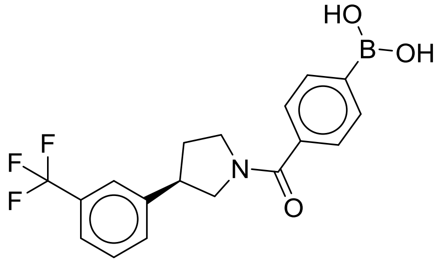	NA
ZINC000584934459	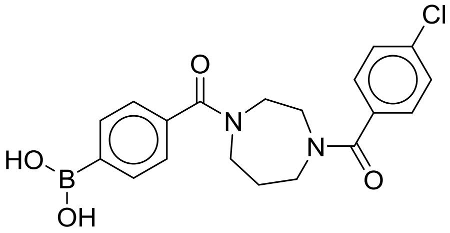	NA
ZINC000584929203	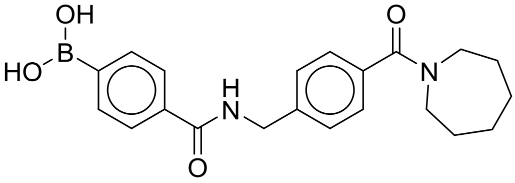	NA
ZINC000584957148	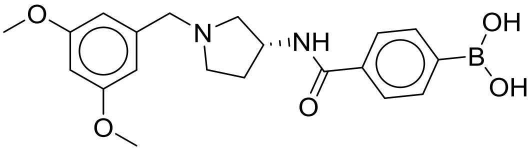	NA
ZINC000584931137	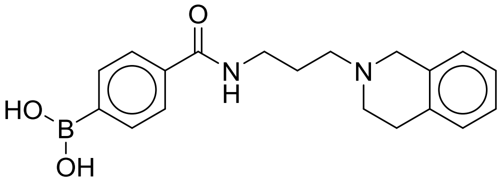	NA
ZINC000584987346	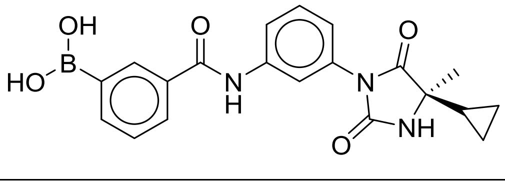	NA

ZINC000584971306	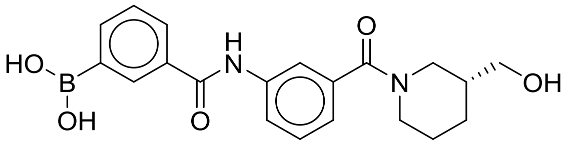	NA
ZINC000584992783	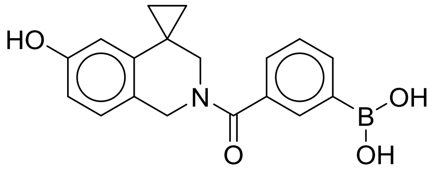	NA
ZINC000585071378	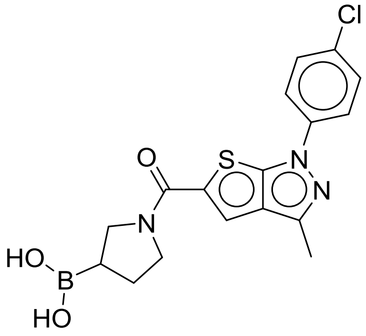	NA
ZINC000585077110 **(3)**	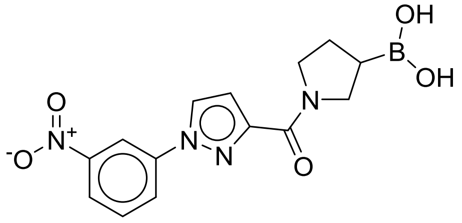	55 ±9
ZINC000585075958	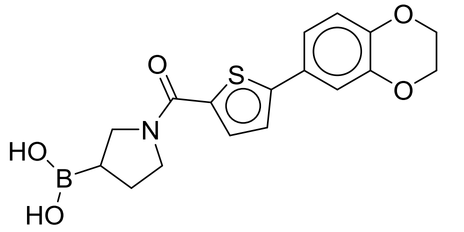	very high background, could not determine RA
ZINC000585076051	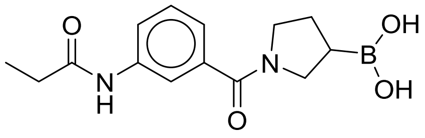	NA
ZINC000585078236 **(1)**	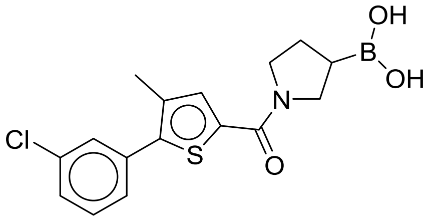	2 ±4
ZINC000585076961 **(4)**	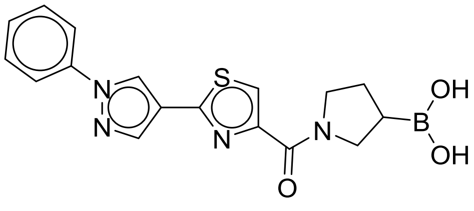	62 ±2

ZINC000585073522<br>**(2)**	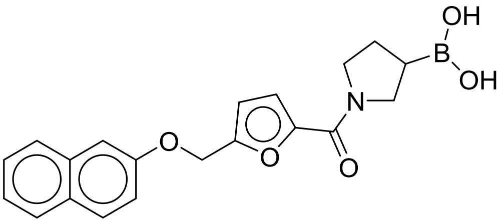	1 ±4

[a] RA, residual activity; NA, not active

References

1. RDKit: Open-source cheminformatics. Release 2019.03.1 (http://www.rdkit.org).
